# Evaluating a team-based approach to research capacity building using a matched-pairs study design

**DOI:** 10.1186/1471-2296-13-16

**Published:** 2012-03-12

**Authors:** Libby Holden, Susan Pager, Xanthe Golenko, Robert S Ware, Robyn Weare

**Affiliations:** 1 School of Medicine, Griffith University, University Drive, Meadowbrook, Queensland 4131, Australia; 2 Queensland Health, Brisbane, Australia; 3 School of Population Health, University of Queensland, Brisbane, Australia

**Keywords:** Research Capacity Building, Research culture, Evaluation, Multi-disciplinary team, Primary health care

## Abstract

**Background:**

There is a continuing need for research capacity building initiatives for primary health care professionals. Historically strategies have focused on interventions aimed at individuals but more recently theoretical frameworks have proposed team-based approaches. Few studies have evaluated these new approaches. This study aims to evaluate a team-based approach to research capacity building (RCB) in primary health using a validated quantitative measure of research capacity in individual, team and organisation domains.

**Methods:**

A non-randomised matched-pairs trial design was used to evaluate the impact of a multi-strategy research capacity building intervention. Four intervention teams recruited from one health service district were compared with four control teams from outside the district, matched on service role and approximate size. All were multi-disciplinary allied health teams with a primary health care role. Random-effects mixed models, adjusting for the potential clustering effect of teams, were used to determine the significance of changes in mean scores from pre- to post-intervention. Comparisons of intervention versus control groups were made for each of the three domains: individual, team and organisation. The Individual Domain measures the research skills of the individual, whereas Team and Organisation Domains measure the team/organisation's capacity to support and foster research, including research culture.

**Results:**

In all three domains (individual, team and organisation) there were no occasions where improvements were significantly greater for the control group (comprising the four control teams, n = 32) compared to the intervention group (comprising the four intervention teams, n = 37) either in total domain score or domain item scores. However, the intervention group had a significantly greater improvement in adjusted scores for the Individual Domain total score and for six of the fifteen Individual Domain items, and to a lesser extent with Team and Organisation Domains (two items in the Team and one in the Organisation domains).

**Conclusions:**

A team-based approach to RCB resulted in considerable improvements in research skills held by individuals for the intervention group compared to controls; and some improvements in the team and organisation's capacity to support research. More strategies targeted at team and organisation research-related policies and procedures may have resulted in increased improvements in these domains.

## Background

Research capacity building (RCB) develops individuals and institutions to higher levels of skill and ability to conduct quality research. It aims to improve the ability to conduct, use and promote research through providing training, funding, infrastructure, linkages and career pathways [1-4]. There is an absence of a critical mass of primary health care researchers, research activity and the ability to carry out quality research [5-7]. This is largely due to lack of research skill, time, funding and linkages to universities [8], and a continued gap between research and practice [9]. A strategic approach to RCB is needed to accommodate the complex and multi-disciplinary context of primary health care (PHC) [10].

Historically RCB interventions have focused on developing the research skills of individuals using strategies such as research skills training, quarantined time for research through Fellowships, writing scholarships, mentoring, journal clubs, and small research seeding grants [1,8,11-15]. Research networks have been another method used to foster research but have historically focused on assisting individual GPs to cultivate an interest in research by providing research skills training and assistance to individuals for their small research project [7,16,17].

More recently a 'whole of system' approach to research capacity building has been proposed which influences different levels of the organisation and allows practitioners to participate at a level that enables them to build on their existing capacity [18]. Cooke provides a framework for conducting and evaluating RCB initiatives with a set of guiding principles to be applied at individual, team, organisation and supra-organisation levels [6]. These principles are to: build skills and confidence, develop linkages and partnerships, ensure research is close to practice, develop appropriate dissemination, invest in infrastructure, and build elements of sustainability/continuity. Similarly, practice-based research networks have been recommended which provide support to research-focused practices rather than individuals [16], and existing networks which target individuals are being encouraged to support a range of practice staff and PHC professionals not just GPs [7]. In order to bring about the culture shift necessary to cultivate quality research in PHC more innovative approaches to RCB are needed [5,10,19,20]; for example, strategies that support PHC teams to conduct research and the 'whole of system' approach described above [5,6,16,18,21].

Although considerable funds have been invested in RCB activities both in Australia and overseas, few studies have rigorously evaluated specific RCB interventions in primary health care [2,8,14,22-24]. These evaluation studies used either the Research Spider [25] to identify improvements in individuals' research skills [14,22], and/or traditional process and output measures such as number of conference presentations and research proposals [8,14]. However, some also developed conceptual frameworks to qualitatively measure the impacts of their intervention [2,23,24].

Only two publications were found that described or evaluated the process of implementing a team or settings based approach to RCB [2,24]. One of these papers provided a qualitative evaluation of the 'Designated Research Team' (DRT) approach, using the six guiding principles for RCB developed by Cooke to evaluate the model [2]. The other paper described the DRT approach using one team as a case study [24]. While tools measuring individual research skill exist, few tools quantitatively identify and measure changes in the organisation's capacity to support research to enable the adequate evaluation of RCB interventions adopting the whole of system approach [26].

This study addresses these gaps in knowledge by providing a team-based approach to RCB for primary health care teams using evidence-based RCB strategies and an evaluation framework using the validated Research Capacity and Culture (RCC) Tool which measures RCB at individual, team and organisation domains [26]. A more detailed description of RCC tool, including its psychometric properties, is provided in the 'Data Collection' section of the Methods. The aim of the study is to evaluate the impacts of an array of RCB initiatives, using a team-based approach to RCB, according to three domains: individual, team and organisation.

## Methods

### Study design

A non-randomised matched-pair trial design was used to compare the impact of a multi-strategy research capacity building (RCB) intervention. Four intervention teams were matched with four control teams on service role and size.

### Study participants and recruitment

Multi-disciplinary PHC teams were recruited from within one geographical district of Queensland Health. Team leaders from multi-disciplinary allied health teams with a primary health care role were approached initially, before a meeting with the team to provide more information. Interested teams applied to participate in the intervention, providing relevant eligibility details. Eligible teams were required to have a PHC focus, have between 5-50 staff, have approval from their manager to participate in the RCB intervention, have an idea for a research project they could develop, and have at least one person in the team with some research experience. As insufficient eligible teams applied to satisfy sample size requirements it was not possible to randomly allocate to intervention or control groups. Therefore suitable control teams were recruited from other districts after matching on size and service role. Control teams completed the same self-report written survey as intervention teams prior to and after the intervention period. No other requirements were placed on controls. Written consent was obtained from individuals in both the intervention and control teams using an informed consent information package. Ethical clearance was obtained from Griffith University and each of the relevant local Queensland Health Ethics Committees.

Variations in team characteristics in relation to the eligibility criteria became evident after baseline data collection. Firstly, preliminary mentoring meetings with teams revealed that teams did not have a research project that was robust and feasible within the intervention time frame. Considerable changes were required to all research project ideas to become viable. Secondly, analysis of the baseline data revealed that most teams did not have a team member with research experience at a level enabling them to mentor others. More often they had experience collecting data for another researcher, not conducting their own ethics-approved research project.

### Intervention program

The 'Designated Research Team' (DRT) approach to RCB described by Cooke provides a documented method similar to that taken in this study. The DRT approach worked with selected teams to support them to conduct a small research project, by providing protected time, skills training and mentoring [24]. Similarly, our approach was to work with selected teams to conduct research relevant to their clinical practice. A comprehensive multi-strategic package of RCB strategies previously documented in the literature [1,8,11-15] was used to develop research capacity and culture within the participating teams. Training and progress review activities were provided to all four teams as one larger group, enabling collaboration, networking and mutual support of projects. Our strategies included: i) specially tailored research skills training programs, timed to coincide with the appropriate phases of their projects and recorded on DVD for those unable to attend in person; ii) ongoing mentoring for each intervention team to support the research project being undertaken as part of the intervention; iii) writing bursaries to support research funding grant applications; iv) some financial support with direct research costs where grant applications were unsuccessful; v) research Fellowships (quarantined time) for one day per week for one person from each team; vi) infrastructure support such as research software, desk and computer use. All teams had good representation at training regarding developing a research idea and project proposal and all had some representation (although the proportion of staff attending varied) at other training events such as writing an ethics application, writing a funding grant application and modules in either qualitative or quantitative methods. Only one team had an experienced internal mentor in their team. This was the only team successful in obtaining external research grant funds for their project, a pilot for a randomized control trial). One other team chose to not apply for a research fellowship or writing bursary as they felt these resources were not required by their team. Financial support provided by the intervention strategy to support the team-based research projects was allocated based on required research costs for each project (ranging from approximately AU$1,000-AU$21,000).

### Data Collection

Data collection for intervention teams occurred in December 2008 (baseline) and March 2010 (follow-up). Baseline and follow-up data collection occurred later for controls (March - June 2009 and May to December 2010); however, the same time period (15 months) between pre- and post-data collection phases occurred for both groups. This delay was due to the matched controls study design and the need to obtain ethical clearance at each district level, once suitable controls had been found.

Tools were specifically designed to measure the effectiveness of this RCB strategy as follows:

i.) A self-report written survey was developed and validated to measure research capacity and culture (RCC Tool) for Organisation, Team and Individual Domains (Cronbach's α = 0.95, 0.96, 0.96 respectively and test-retest reliability of intra-class correlations = 0.77, 0.83, and 0.82 respectively) [26]. For the Organisation and Team Domains the respondent ranks their organisation's or team's success/skill level for all 20 items in each domain. Each item has a response scale of 1-10 with 1 being least success/skill and 10 the highest possible success/skill level. The same response scale system applies to the individual domain where the respondent ranks his or her own success/skill level on 15 items. Data were also collected on research outputs but were not included in the analysis due to poor test-retest reliability of these indicators.

The following qualitative data (items ii) and iii) below) were collected for the purpose of providing contextual information to support the interpretation of the research findings in the discussion section of this paper. A separate paper (unpublished) reports findings from Senior Managers.

ii.) Team leaders from participating teams completed a written survey both pre- and post-intervention to identify changes in team structure, role and management that may impact on the study.

iii.) Senior managers of both intervention and control teams were interviewed both pre- and post-intervention to provide contextual information to identify organisational factors that could impact on the evaluation of the intervention.

### Statistical analysis

A minimum number of four people in each of the intervention teams and four in each of the control teams was required and an overall sample of 16 in the intervention group and 16 in the control group was required to demonstrate a change of 15% with a power of 80%, a significance level of 0.05 and a standard deviation of 40. We used linear regression analysis to determine if there were significant differences in baseline domain total scores between intervention and control groups. We adjusted for potential within-team clustering by including variables identifying teams and individuals within teams in our analytic models. For each of the Individual, Team, and Organisation Domains we report the pre- and post-intervention mean for each item. To determine if the mean scores changed significantly over time when comparing control group and intervention group we used a random-effects mixed model. This type of model adjusts for the potential clustering effect of individuals within teams and enables missing data to be intrinsically imputed for those with a baseline score but no follow-up score. Models included time and treatment group as the main effects and time by treatment as the interaction term, and models were performed with and without adjusting for highest education level. Models were analysed with and without adjusting for highest level of qualification as it was thought that formal training may be a potential confounder influencing outcomes. Statistical analysis was performed in Stata v11.2 (Statacorp, College Station, TX, USA).

## Results

A total of 69 primary health care professionals from multi-disciplinary teams (four intervention and four control teams) were recruited to the study. Table 1 reports the profession and highest qualification at baseline for each group. Only one intervention team had medical staff (2 Paediatricians) and the matched control team had one Paediatrician. No medical students were part of teams. Few differences were detected between groups for these characteristics. There were no statistically significant differences at baseline between control and intervention groups in total scores for each of the three domains (organisation, team and individual) (Table 2).

**Table 1:** Baseline Profile of Sample

	Controls n = 32	Interventions n = 37
**Profession**		

Allied health Assistant	0	1

Dieticians	0	1

Occupational Therapists	8	9

Physiotherapists	8	8

Speech Pathology/audiologists	6	8

Social workers	1	4

Psychologists	1	2

Other: nurses, doctors, health promotion	6	4

**Highest qualification**		

Tafe	0	1

Undergraduate	23	28

Postgraduate	7	8

PhD	1	0

Missing: Profession = 2; highest qualification = 1		

**Table 2:** Comparison of baseline domain scores^# ^for intervention group compared to control group to determine if any significant differences between groups prior to intervention (using linear regression adjusting for teams and individuals)

	Control group (n = 32)		Intervention group (n = 37)			
	**Baseline** **mean**	**Inter-quartile range**	**Baseline** **mean**	**Inter-quartile range**	**Coefficient**	**p value**

Organisational domain	5.70	3.85-7.00	5.69	4.03-7.07	-0.24	0.79

Team domain	5.53	4.04-7.17	5.58	3.91-7.08	-1.07	0.30

Individual domain	4.65	2.93-5.97	4.13	2.57-5.6	-1.45	0.16

* = significant difference at p < 0.05; ** = highly significant difference at p < 0.001; † = trend of < 0.1

# Tables 3, 4, and 5 list the items included in each of these domains. Scores on these items are added to calculate the total score for each domain

Table 3 displays the domain scores in the Individual Domain. There was a significantly greater improvement in the total domain scores in the Individual Domain and in six of the 15 items in that domain for the intervention group compared to the control group. These items related to writing a research protocol, securing research funding, submitting an ethics application, analysing qualitative data, writing a research report, providing advice to less experienced researchers and a trend only for writing for publication.

**Table 3:** INDIVIDUAL DOMAIN item and total scores for Control group compared with Intervention group (using hierarchical modelling to adjust for the potential clustering effect of individuals within teams)

	Control Group Individuals (n = 32)		Intervention Group Individuals (n = 37)		Unadjusted significance		Adjusted for highest qualification at baseline	
**Individual Domain**	**Mean baseline**	**Mean follow-up**	**Mean baseline**	**Mean** **follow-up**	**Coefficient**	**P value**	**Coefficient**	**P value**

i) Finding relevant literature	6.42	6.89	6.00	7.59	0.72	0.16	0.87	0.10

ii) Critically reviewing the literature	6.03	6.72	5.78	6.69	-0.33	0.95	0.25	0.61

iii) Using a computer referencing system (eg Endnote)	4.52	5.22	3.47	4.71	0.59	0.38	0.54	0.43

iv) Writing a research protocol	4.06	4.39	3.67	5.69	1.42	< 0.05*	1.76	< 0.05*

v) Securing research funding	2.90	3.29	2.75	4.70	1.53	< 0.05*	1.46	< 0.05*

vi) Submitting an ethics application	3.48	3.47	3.58	6.10	2.38	< 0.001**	2.37	< 0.001**

vii) Designing questionnaires	5.00	5.67	4.39	5.96	0.61	0.38	0.86	0.23

viii) Collecting data e.g. surveys, interviews	5.58	6.72	5.53	7.00	0.22	0.76	0.42	0.57

ix) Using computer data management systems	4.16	5.22	3.71	4.68	-0.39	0.93	0.11	0.81

x) Analysing qualitative research data	4.39	5.47	2.97	5.10	1.14	< 0.1^†^	1.27	< 0.05*

xi) Analysing quantitative research data	3.94	5.17	3.67	4.39	-0.54	0.38	-0.40	0.53

xii) Writing a research report	4.45	4.39	4.00	5.18	0.96	< 0.1^†^	1.28	< 0.05*

xiii) Writing for publication in peer-reviewed journals	3.81	4.00	3.14	4.80	1.15	< .0.05*	1.04	< 0.1^†^

xiv) Integrating research findings into practice	6.34	7.11	6.28	6.76	-0.27	0.67	-0.18	0.79

xv) Providing advice to less experienced researchers	6.67	3.76	3.00	4.93	1.22	< 0.05*	1.23	< 0.05*

**Total score**	4.65	5.19	4.13	5.63	0.98	< 0.05*	1.11	< 0.05*

* = significant difference at p < 0.05; ** = highly significant difference at p < 0.001; † = trend of < 0.1

Table 4 displays the domain scores in the Team Domain. There was no significant difference in total domain score change between the intervention and control groups but there were for the following Team Domain items: "Has consumer involvement in research activities/planning"; and "Has external partners (e.g. universities) engaged in research"; and a trend of p < 0.1 for "Provides resources to support staff research training".

**Table 4:** TEAM DOMAIN item and total scores for Control group compared with Intervention group (using hierarchical modelling to adjust for the potential clustering effect of individuals within teams)

	Control Group Individuals (n = 32)		Intervention Group Individuals (n = 37)		Unadjusted significance		Adjusted for highest qualification at baseline	
**Team Domain**	**Mean baseline**	**Mean follow-up**	**Mean baseline**	**Mean** **follow-up**	**Coefficient**	**P value**	**Coefficient**	**P value**

i) provides resources to support staff research training	4.25	4.83	4.09	6.10	1.02	< 0.1^†^	1.04	< 0.1^†^

ii) has funds, equipment or admin to support research activities	3.87	4.35	3.45	4.89	0.80	0.22	0.90	0.18

iii) does team level planning for research development	4.68	5.72	4.85	6.00	-0.02	0.98	0.05	1.00

iv) ensures staff involvement in developing that plan	5.47	6.94	5.14	6.38	-0.37	0.67	-0.22	0.81

v) has team leaders that support research	7.20	7.71	7.72	8.57	0.21	0.77	0.45	0.53

vi) provides opportunities to get involved in research	5.48	6.47	6.53	8.07	0.55	0.51	0.77	0.36

vii) does planning that is guided by evidence	6.87	7.94	6.14	7.17	-0.13	0.86	0.10	0.90

viii) has consumer involvement in research activities/planning	5.31	5.13	4.30	5.59	1.44	< 0.1^†^	1.51	< 0.05*

ix) has applied for external funding for research	5.57	5.86	6.30	7.04	0.51	0.63	0.87	0.41

x) provides access to literature searching and article retrieval	6.84	7.78	6.47	7.28	-0.13	0.84	-0.17	0.81

xi) conducts research activities relevant to practice	5.77	7.22	5.83	7.66	0.25	0.78	0.42	0.64

xii) supports applications for research scholarships/degrees	6.33	6.73	6.13	6.96	0.35	0.72	0.70	0.48

xiii) has mechanisms to monitor research quality	5.04	5.71	4.26	5.92	0.94	0.29	1.25	0.17

xiv) provides access to experts for research advice	5.12	5.53	5.71	7.52	1.07	0.23	1.20	0.20

xv) disseminates research results at research forums/seminars	5.52	8.88	5.2	6.26	0.66	0.38	1.02	0.17

xvi) supports a multi-disciplinary approach to research	6.81	7.44	6.94	8.38	0.86	0.26	1.14	0.14

xvii) has incentives & support for mentoring activities	5.21	5.31	4.48	5.39	0.84	0.28	0.90	0.26

xviii) has external partners (eg universities) engaged in research	5.11	5.53	4.56	7.66	2.40	< 0.05*	2.63	< 0.05*

xix) supports peer-reviewed publication of research	4.43	6.13	4.85	7.00	0.78	0.29	0.88	0.25

xx) provides access to software to support research activities	3.69	4.79	2.96	5.56	0.21	0.82	0.34	0.73

**Total score**	5.53	6.24	5.79	6.72	0.38	0.50	0.53	0.36

* = significant difference at p < 0.05; ** = highly significant difference at p < 0.001; † = trend of < 0.1

Table 5 displays the domain scores in the Organisation Domain. There was no significant difference in total domain score but there were for the Organisation Domain item: "Supports a multi-disciplinary approach to research"; and trends of p < 0.1 for Organisation Domain items: "Provides adequate resources to support staff research training" and "Has funds, equipment or admin to support research".

**Table 5:** ORGANISATION DOMAIN item and total scores for Control group compared with Intervention group (using hierarchical modelling to adjust for the potential clustering effect of individuals within teams)

	Control Group Individuals (n = 32)		Intervention Group Individuals (n = 37)		Unadjusted significance		Adjusted for highest qualification at baseline	
**Organisational Domain**	**Mean baseline**	**Mean follow-up**	**Mean baseline**	**Mean** **follow-up**	**Coefficient**	**P value**	**Coefficient**	**P value**

i) provides adequate resources to support staff research training	4.93	4.83	4.77	5.90	0.98	0.12	1.13	< 0.1^†^

ii) has funds, equipment or admin to support research activities	4.33	4.33	4.10	5.36	1.05	0.12	1.17	< 0.1^†^

iii) has a plan or policy for research development	4.82	5.38	5.50	6.39	0.22	0.80	0.33	0.71

iv) provides access to literature search and article retrieval	6.70	7.56	6.69	6.79	-0.69	0.36	-0.68	0.37

v) has senior managers that support research	6.59	6.56	6.97	7.48	0.45	0.52	0.60	0.38

vi) ensures staff career pathways are available in research	4.41	4.94	4.72	5.52	0.20	0.78	0.25	0.73

vii) ensures organisation planning is guided by evidence	6.12	6.67	5.29	5.54	-0.35	0.57	-0.12	0.66

viii) has consumers involved in research	5.24	4.73	4.92	5.52	1.07	0.27	0.91	0.37

ix) accesses external funding for research	5.32	5.06	5.59	6.15	0.93	0.25	1.11	0.18

x) promotes clinical practice based on evidence	7.07	7.78	7.14	7.43	-0.20	0.74	-0.22	0.72

xi) encourages research activities relevant to practice	6.17	6.33	6.06	6.93	0.62	0.33	0.67	0.31

xii) provides access software for analysing research data	4.47	4.38	3.00	4.77	1.05	0.25	1.01	0.29

xiii) has mechanisms to monitor research quality	4.60	4.86	3.91	5.00	0.57	0.53	0.36	0.70

xiv) provides access to experts for research advice	4.57	4.76	4.97	6.55	0.98	0.21	1.01	0.21

xv) supports a multi-disciplinary approach to research	6.27	6.00	6.09	7.34	1.50	< 0.05*	1.43	< 0.05*

xvi) provides forums/bulletins to present research findings	4.48	4.59	4.61	5.11	0.14	0.85	0.03	0.97

xvii) engages external partners (eg universities) in research	5.44	5.94	5.67	7.10	1.01	0.13	1.13	0.10

xviii) supports applications for research scholarships/degrees	5.61	6.39	5.52	7.25	0.94	0.21	1.02	0.19

xix) supports the peer-reviewed publication of research	5.30	6.19	5.50	6.83	0.08	0.90	0.27	0.68

xx) requires ethics approval for research activities	8.27	8.71	8.32	9.10	0.18	0.77	0.45	0.46

**Total score**	5.70	5.85	5.69	6.47	0.68	0.15	0.73	0.14

* = significant difference at p < 0.05; ** = highly significant difference at p < 0.001; † = trend of < 0.1

## Discussion

This study provides empirical evidence of the effectiveness of a team-based approach to RCB in primary health care. Statistically significant improvements were observed for the intervention group compared to the control group within the Individual Domain (which measures research skills and competences held by the individual) for both the total domain score and for six of the fifteen Individual Domain items. It should be noted, however, that even where there was a significant improvement in scores the actual scores generally remained low for both groups; indicating an ongoing need for RCB support for allied health professionals. Similarly, for the Team and Organisation Domains (which measure the team/organisations capacity to support research and to provide a culture that promotes research) there was no significant improvement in total domain scores but there were for two of the Team Domain items and one of the Organisation Domain items; with additional items demonstrating trends of p < 0.1 (one in the Organisation Domain and two in the Team Domain). In all three domains there were no occasions where improvements were significantly greater for the control group compared to the intervention group.

These findings demonstrate the RCB intervention targeted at teams results in considerable improvements in the research skills held by individuals within those teams and, to a lesser extent, improvements in the teams' and the organisation's capacity to support research and to provide a culture that fosters research. We believe this reduced impact on team and organisation domains was largely due to the type of interventions conducted within this RCB strategy. That is, strategies were targeted at the development of research capacity through skill development and research activity but not directly targeted at changing policy and practice of teams or the organisation. However, a lack of research champions/mentors within teams could also have hindered the development of team-level indicators of research culture. This lack of capacity is reflected across allied health and primary health care [5-7]. Strategies to improve capacity have been identified recently in a study by Pager et. al. Identified strategies include working with staff already motivated to do research and providing them with time and funds to conduct research, opportunities for career advancement linked to research, access to mentors and links to universities [27].

Strengths of the study include the use of a control group, which provides a reference for change that may have occurred regardless of the intervention. The use of a validated tool to quantitatively measure research capacity in all three domains adds to the rigor of the evaluation. The fact the intervention occurred within one large organisation only, not several organisations, limits the generalizability of our findings. However, it also provides the same broad contextual environment for both intervention and control groups which could be beneficial. Because insufficient teams applied to participate in the study we were not able to randomise participation to intervention or control groups. This resulted in a matched-controls design with a time lag between groups, which could dilute the effect of the intervention as the organisation may be further progressed in implementing its research development strategy than when data collection occurred for intervention teams. A larger sample size is likely to have resulted in more items demonstrating statistical significance as many items showed a greater increase for interventions than controls but did not reach significance. This could indicate that our study may be underpowered to detect modest, but important, differences. Due to funding limitations the duration of the intervention was less than desirable to bring about change [28]. A two year intervention is likely more realistic to support a team of novice researchers from inception to manuscript submission stage in the research process.

This RCB initiative occurred within an environment of considerable change. Interviews with senior managers of both intervention and control groups at pre- and post-intervention identified that during the intervention period of 15 months there was an organisation restructure that involved realignment of allied health divisions within the organisation to match new district geographical boundaries. Other changes during that period identified by senior managers were the on-going negotiation of a new pay award, an increased demand on services, and a resource-intensive RCB initiative undertaken by the organisation. Senior managers expressed hope that the award restructure and organisational RCB strategy would provide additional opportunity to support and recognise research; and that the realignment of divisions, along with the establishment of joint research appointments and research centres would result in greater critical mass to conduct research. They reported that unfortunately some of these strategies, in the short term at least, have resulted in unstable ground and increased work load. For example, the slowing down of systems and processes, such as getting ethical clearance; and funding allocation that does not benefit all causing dissention among staff.

At a team or service level, written surveys completed by team leaders at pre- and post-intervention for both intervention and control groups identified high levels of staff turn-over, including management staff, during the intervention period. Team leaders also reported increased workload demands without increased staffing levels, and significant changes impacting on services and staff resulting from the organisation restructure. There were no differences in circumstances for intervention teams compared with controls with the exception that two intervention teams also experienced a new model of care which resulted in a considerably increased workload. This added workload is likely to have negatively impacted on the potential for these teams to participate in the RCB activities and thus diminished potential improvements to RCB available through the initiatives. Similarly, this changing environment brings challenges to the process of supporting teams of novice researchers to conduct research; therefore, consideration to the amount and complexity of change the teams and individuals can cope with needs to be given. This context is not uncommon according to Rosenbeck, 2001 who sees organisational processes as a missing link between research and practice, stating that "complex organisations are characterised by multiple competing goals and fluid involvement of key participants". He emphasises the importance of developing self-sustaining communities of practice within these organisations [21].

The areas in which our study found significant improvements for intervention teams in the Organisation and Team Domains are consistent with those reported in the evaluation of the DRT approach [2]. Cooke et.al, 2008, found a team-based approach effective in developing linkages, collaborations and skills [2]. Similarly, our study found significantly improved resources for skills development and research, improved support for multi-disciplinary research, improved involvement of consumers in research and improved links to external partners such as universities. External linkages have also been reported as important for supporting practice-based research [29]. At an individual level our study found significant improvements in a range of indicators of research skill or capacity, particularly related to research activity skills rather than evidence based practice related skills.

Factors contributing to the success of the research and RCB outcomes for our study are consistent with those described for the DRT approach. Protected time, managerial support, applied timely research skills training, mentorship, critical mass of research experience in teams and an encouraging work environment were all identified as important factors of success [24]. Our intervention included all of these characteristics although, unfortunately there were not experienced researchers in our teams; therefore, no critical mass at baseline, as was the case for the DRT teams.

Some teams had not completed their projects by the time post-intervention data collection occurred. These teams continued on with their projects to the write up stage with minimal support, demonstrating a potential for sustainability beyond the scope of the program. This was consistent with findings from the DRT evaluation which reported that research activity was sustained beyond the intervention period and that a research question derived from clinical practice was an important factor [24].

The greatest impact of our intervention was seen in the Individual Domain. This is largely because the intervention focused more on skill development and applied research activity rather than changes to policy and practice. The feedback meetings conducted for formative evaluation during the intervention period clearly indicated teams were at their maximum capacity due to heavy workloads, so their ability to take on additional tasks such as skill development, research activity or changes to policies and infrastructure to support research was limited. This was due in part to the additional workload generated from organisational change factors. Therefore we focussed on interventions that supported teams to conduct the research project they had committed to. Feedback also highlighted the importance of having sufficient lead time working with teams to develop a viable research project with agreed tasks and time lines, and sufficient supported time to conduct the research activities with RCB support, such as research skills training at appropriate times. The complexity of conducting research in teams revealed additional strengths and challenges to the research process. For example, shared task management and research vision were strengths whereas decision-making and making time where clinicians were available for collaboration was difficult amid heavy clinical loads. Future directions in research to evaluate team-based interventions for RCB would benefit from designing a study with the following characteristics: a randomised intervention and control design using separate organisations for each team and targeting interventions to include refining research related policies and practices; a larger sample; longer intervention period and longer-term follow-up evaluation. The lack of strategies targeted at team and organisation levels are a limitation of this intervention design. However, a longer intervention period would be required to support this as teams indicated they did not have the capacity to take on additional activities beyond conducting their research project and the skills development required to support that.

We found that a team-based approach to RCB was effective for novice researchers with demanding clinical loads. The approach enabled clinicians to conduct a research project close to clinical practice and to share the responsibilities of the research among the group. The approach also enabled RCB implementers to support considerably more people to get experience in research activity than would be possible if supporting individuals each working on a separate project. With a continued need for developing research culture and capacity in PHC this study provides valuable evidence in support of an innovative and efficient approach to RCB.

## Conclusions

A team-based approach to RCB is an effective and innovative method of developing research culture and capacity in PHC.

## Competing interests

The authors declare that they have no competing interest.

## Authors' contributions

Each of the authors made a significant contribution to the conception and design, and/or data analysis and interpretation for the study. All authors have contributed to and reviewed the draft and final versions of this manuscript.

## Pre-publication history

The pre-publication history for this paper can be accessed here:

http://www.biomedcentral.com/1471-2296/13/16/prepub
